# Bat Astrovirus in Mozambique

**DOI:** 10.1186/s12985-018-1011-x

**Published:** 2018-06-20

**Authors:** Flora Hoarau, Gildas Le Minter, Léa Joffrin, M. Corrie Schoeman, Erwan Lagadec, Beza Ramasindrazana, Andréa Dos Santos, Steven M. Goodman, Eduardo S. Gudo, Patrick Mavingui, Camille Lebarbenchon

**Affiliations:** 1 Université de La Réunion, UMR Processus Infectieux en Milieu Insulaire Tropical (PIMIT), INSERM 1187, CNRS 9192, IRD 249, 2 rue Maxime Rivière (GIP CYROI), 97490 Sainte-Clotilde, La Réunion France; 2 School of Life Sciences, Biological Sciences Building, University of Kwa-Zulu Natal, South Ring Road, Westville Campus, Kwa-Zulu Natal, 3630 South Africa; 3 Association Vahatra, BP 3972, 101 Antananarivo, Madagascar; 4 Veterinary Faculty, Eduardo Mondlane University, Maputo, Mozambique; 5 Field Museum of Natural History, 1400 South Lake Shore Drive, Chicago, 60605 USA; 6 Instituto Nacional de Saúde, Maputo, Mozambique; 7 Present Address: Institut Pasteur de Madagascar,Ambatofotsikely, 101 Antananarivo, Madagascar

**Keywords:** Mammastrovirus, Mayotte, Mozambique, Madagascar, *Triaenops afer*

## Abstract

Astroviruses (AstVs) are responsible for infection of a large diversity of mammalian and avian species, including bats, aquatic birds, livestock and humans. We investigated AstVs circulation in bats in Mozambique and Mayotte, a small island in the Comoros Archipelago located between east Africa and Madagascar. Biological material was collected from 338 bats and tested for the presence of the AstV RNA-dependent RNA-polymerase gene with a pan-AstV semi-nested polymerase chain reaction assay. None of the 79 samples obtained from Mayotte bats (*Pteropus seychellensis comorensis* and *Chaerephon pusillus*) tested positive; however, 20.1% of bats sampled in Mozambique shed AstVs at the time of sampling and significant interspecific variation in the proportion of positive bats was detected. Many AstVs sequences obtained from a given bat species clustered in different phylogenetic lineages, while others seem to reflect some level of host-virus association, but also with AstVs previously reported from Malagasy bats. Our findings support active circulation of a large diversity of AstVs in bats in the western Indian Ocean islands, including the southeastern African coast, and highlight the need for more detailed assessment of its risk of zoonotic transmission to human populations.

**Electronic supplementary material:**

The online version of this article (10.1186/s12985-018-1011-x) contains supplementary material, which is available to authorized users.

Astroviruses (AstVs) are small non-enveloped RNA viruses, transmitted via the fecal-oral route. They have been detected from over 80 vertebrate host species [1], and represent a significant source of morbidity and economic losses. Worldwide, AstVs account for 2 to 9% of all acute non-bacterial gastroenteritis in children [2]; they are also responsible for diseases in livestock, poultry and domestic pets [3]. In wild animals, AstVs have been mostly detected in bats [4] and in aquatic birds [5], although detection in other host types have been reported, such as in marine mammals [6] and non-human primates [7].

Current knowledge on the epidemiology of AstVs in African bats is limited [8, 9]. In a previous study, we detected high genetic diversity of AstVs in Malagasy bats [10]. Detection of AstVs on other islands of the western Indian Ocean has not been reported. The goal of this study was to investigate AstV circulation in bats in Mozambique and on Mayotte, a small island in the Comoros archipelago located between east Africa and Madagascar.

Biological material was collected on Mayotte at several locations (Bandrele, Chiconi, Coconi, Kwale, Mangajou, Passamainty, Sohoabe, Tsoundzou), in November–December 2014, and in Mozambique (Inhassoro district) in February and May 2015. Bats were captured using mist nets and harp traps. On Mayotte, rectal swabs were obtained with sterile rayon-tipped applicators (Puritan, Guilford, ME, USA) from 21 *Pteropus seychellensis comorensis*, and droppings were collected from 58 *Chaerephon pusillus.* Swabs and droppings were placed in 1.5 mL of Virus Transport Media (VTM; [10]), and were immediately frozen in liquid nitrogen. In Mozambique, one rectal and one buccal swab were collected for each sampled bat. The two swabs were then placed in the same tube, containing 1.5 mL of VTM, and were immediately frozen in liquid nitrogen. Sampled bat species and number of tested samples are presented in Table 1.

**Table 1:** Family, species day roosts, and number of bats sampled and tested for the presence of Astroviruses, in Mozambique

Family	Species	Day roosts	N tested	N positive
Hipposideridae	*Hipposideros caffer*	Caves	57	10
Miniopteridae	*Miniopterus mossambicus*	Caves	21	2
Molossidae	*Mops condylurus*	Houses	52	1
Nycteridae	*Nycteris thebaica*	Caves	14	4
Rhinolophidae	*Rhinolophus lobatus*	Caves	9	0
	*Rhinolophus mossambicus*	Caves	20	0
	*Rhinolophus rhodesiae*	Caves	31	0
Rhinonycteridae	*Triaenops afer*	Caves	51	35
Vespertilionidae	*Neoromicia nana*	Rolled-up banana leaves	2	0
	*Scotophilus viridis*	Free-flying	2	0

RNA extraction was performed with the QIAamp Viral RNA Mini Kit (QIAGEN, Valencia, CA, USA). Reverse transcription was performed on 10 μL of RNA using the ProtoScript II Reverse Transcriptase and Random Primer 6 (New England BioLabs, Ipswich, MA, USA) using a previously published protocol [10]. cDNAs were tested for the presence of the AstV RNA-dependent RNA-polymerase (RdRp) gene using a pan-AstV semi-nested polymerase chain reaction (PCR) assay [10, 11]. PCRs were performed with the GoTaq G2 Hot Start Green Master Mix (Promega, Madison, WI, USA) in an Applied Biosystems 2720 Thermal Cycler (Thermo Fisher Scientific, Waltham, MA, USA). Electrophoresis were performed on 1.5% agarose gels stained with 2% GelRed (Biotium, Hayward, CA, USA). Chi square tests were conducted to investigate the effect of the host species, sampling period (month), and sex, on the probability of successful detection of AstV RdRp genes. Statistical analyses were conducted with R, version 3.2.3 [12].

PCR products of the expected size were submitted for direct Sanger sequencing (Genoscreen, Lille, France). The 31 sequences obtained in this study were aligned with 112 reference AstV RdRp partial nucleotide sequences, with CLC Sequence Viewer version 7.7.1 (CLC Bio, Aarhus, Denmark). A maximum-likelihood analysis was performed Phylogenetic trees were constructed by maximum likelihood with the software PhyML 3.1 [13]. The evolutionary model was selected by Model Generator 0.85 (GTR + I + Г, *I* = 0.10, α = 0.71; [14]), and nodal supports were assessed with 1000 bootstrap replicates. A Bayesian Markov Chain Monte Carlo coalescent analysis was also performed, with the program BEAST, version 1.8.4 [15], and the Shapiro-Rambaut-Drummond-2006 (SRD06) nucleotide substitution model [16]. A strict molecular clock and a constant population size were selected. The analysis was performed with a chain length of 60 million generations sampled every 1000 iterations, with first 10% trees discarded as burn-in. The maximum clade credibility tree was visualized with FigTree, version 1.4.3 (http://tree.bio.ed.ac.uk/software/figtree).

None of the 79 samples collected on Mayotte tested positive for the presence of AstV. Although this negative result may be affected by the relatively small sample size and differences in sampling protocols (swabs vs droppings), it may also suggest temporal variation in AstVs shedding and circulation in bat populations, as previously documented [17]. Additional studies are thus needed before concluding that AstVs do not circulate in Mayotte bats.

In Mozambique, 52 of the 259 bats tested positive for the presence of AstV RdRp (mean detection rate ± 95% confidence interval: 20.1% ± 4.9%). This detection rate was similar to other studies using the same PCR assay, including the one we reported on Malagasy bats (22.5% ± 6.1%; [10]). For the Mozambique samples, five of the ten bat species tested positive (Table 1 and Additional file 1 for details), with significant variation between species (*χ*^2^ = 104, *P* < 0.001). A high detection rate was found in *Triaenops afer* (68.6% ± 12.7%), as compared to other species (Table 1). Significant variation was also found between the two sampling sessions (*χ*^2^ = 9, *P* < 0.005) with a higher detection rate in May (25.3% ± 6.5%) than in February (10.1% ± 6.3%), in particular for *T. afer* (*χ*^2^ = 13, *P* < 0.001; 20% ± 24.7% in February, and 80.5% ± 12.1% in May). This variation may be associated with factors related with bat population dynamics facilitating or limiting virus transmission (e.g. population size, density, age structure, body condition [18, 19]). No significant difference was found in AstV detection rate between males and females (*χ*^2^ = 0.4, *P* < 0.5).

High genetic diversity was detected among AstVs sequences obtained from Mozambican bats (pairwise distance up to 45%), without strong support for host family or species restriction (Fig. 1 and Additional file 2 for details), as commonly described for bat AstVs [8, 10, 11, 20, 21]. Most of the detected viruses clustered in large phylogenetic lineages, in particular for *Triaenops afer* and *Hipposideros caffer*, although statistical support was limited. Sequences of AstVs detected in *Nycteris thebaica* and *Mops condylurus* were mostly highly divergent and not included in larger genetic lineages comprising viruses of same bat family or the same geographic area (Fig. 1).

**Fig. 1:**
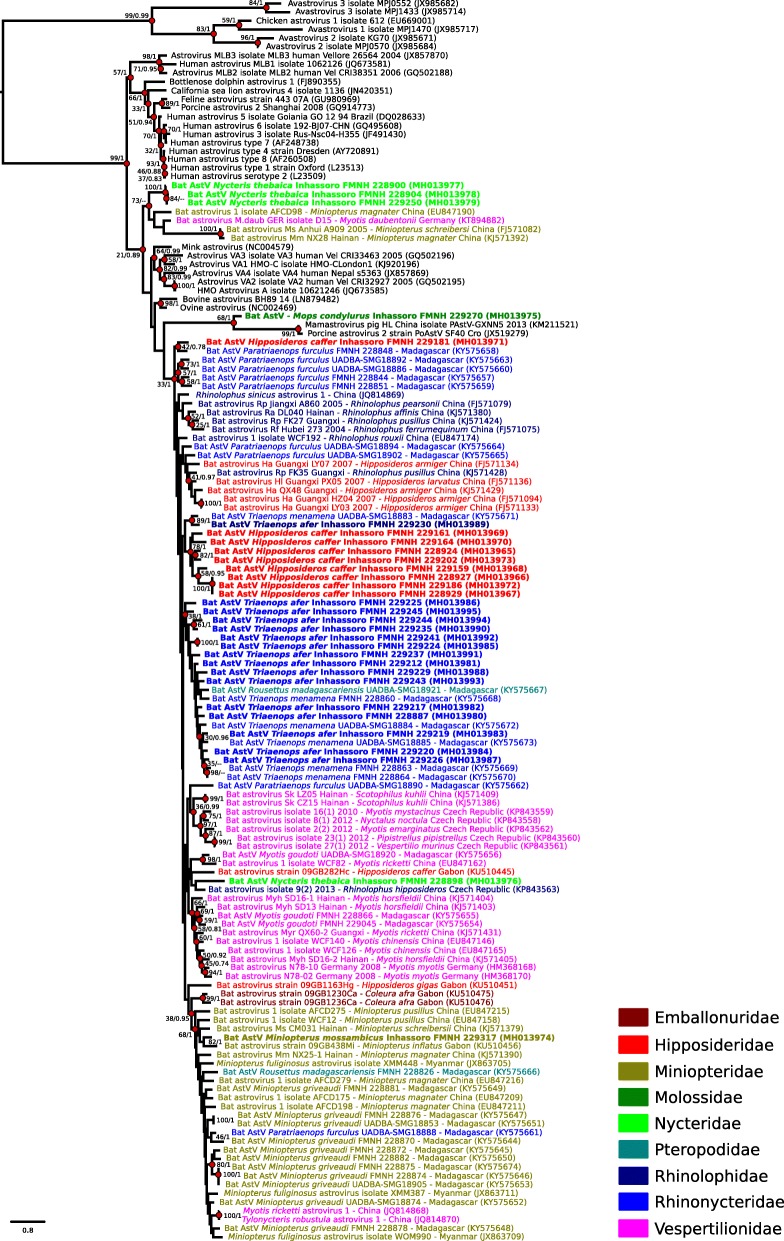
Maximum Likelihood (ML) consensus tree derived from 143 Astrovirus (AstV) RNA-dependent RNA-polymerase partial nucleotide sequences (380 bp). Colored circles indicate nodes with bootstrap values > 70 in the ML tree, or posterior probabilities higher than 0.7 in the maximum clade credibility tree. Sequence names in bold indicate bat AstVs detected in this study, and were colored according to the bat family. Scale bar indicates mean number of nucleotide substitutions per site

The limited genetic information available for AstVs in public databases [1], as well as the high saturation of their genome [22], considerably affects the resolution of phylogenetic trees. Current understanding of the long-time evolutionary history of *Astroviridae* therefore remains limited. In addition, ecological factors involved in AstV infection in bats need to be better assessed. High temporal dynamics of viral infection has been documented before [17], and the risk of spillover to other hosts, including humans, has also been demonstrated to coincide with changes in bat behavior and population structure [23]. The high propensity of AstVs for host shifts highlight the need for a better assessment of zoonotic transmission risk to human populations, particularly in relationship to some unique aspects of bat immunology and ecology.

## Additional files

Additional file 1: Detailed results of Astrovirus detection in samples from Mozambique. (ODS 30 kb)

Additional file 2: List of the bat families and species included in the phylogenetic tree. (PDF 44 kb)

## Abbreviations

AstV: Astrovirus
PCR: Polymerase chain reaction
RdRp: RNA-dependent RNA-polymerase
VTM: Virus Transport Media
