# Supplementary material for: Bat Astrovirus in Mozambique
Source: Virol J. 2018 Jun 20;15:104. doi: 10.1186/s12985-018-1011-x (PMC6011250; doi:10.1186/s12985-018-1011-x)
Supplement: Supplementary file 2 — List of the bat families and species included in the phylogenetic tree. (PDF 44 kb) [file 12985_2018_1011_MOESM2_ESM.pdf]

Family	Species
Emballonuridae	*Coleura afra*
Hipposideridae	*Hipposideros armiger*
	Hipposideros caffer
	Hipposideros gigas
	Hipposideros larvatus
Miniopteridae	*Miniopterus fuliginosus*
	Miniopterus griveaudi
	Miniopterus inflatus
	Miniopterus magnater
	Miniopterus mossambicus
	Miniopterus pusillus
	Miniopterus schreibersii
Molossidae	*Mops condylurus*
Nycteridae	*Nycteris thebaica*
Pteropodidae	*Rousettus madagascariensis*
Rhinolophidae	*Rhinolophus affinis*
	Rhinolophus ferrumequinum
	Rhinolophus hipposideros
	Rhinolophus pearsonii
	Rhinolophus pusillus
	Rhinolophus rouxii
	Rhinolophus sinicus
Rhinonycteridae	*Paratriaenops furculus*
	Triaenops afer
	Triaenops menamena
Vespertilionidae	*Myotis chinensis*
	Myotis daubentonii
	Myotis emarginatus
	Myotis goudoti
	Myotis horsfieldii
	Myotis myotis
	Myotis mystacinus
	Myotis ricketti
	Nyctalus noctula
	Pipistrellus pipistrellus
	Scotophilus kuhlii
	Tylonycteris robustula
	Vespertilio murinus
